# Supplementary figures and images for: Karst-environments of the southeastern Yucatan Peninsula: Hotspots for modern freshwater microbialites
Source: PLoS One. 2025 May 7;20(5):e0322625. doi: 10.1371/journal.pone.0322625 (PMC12057922; doi:10.1371/journal.pone.0322625)

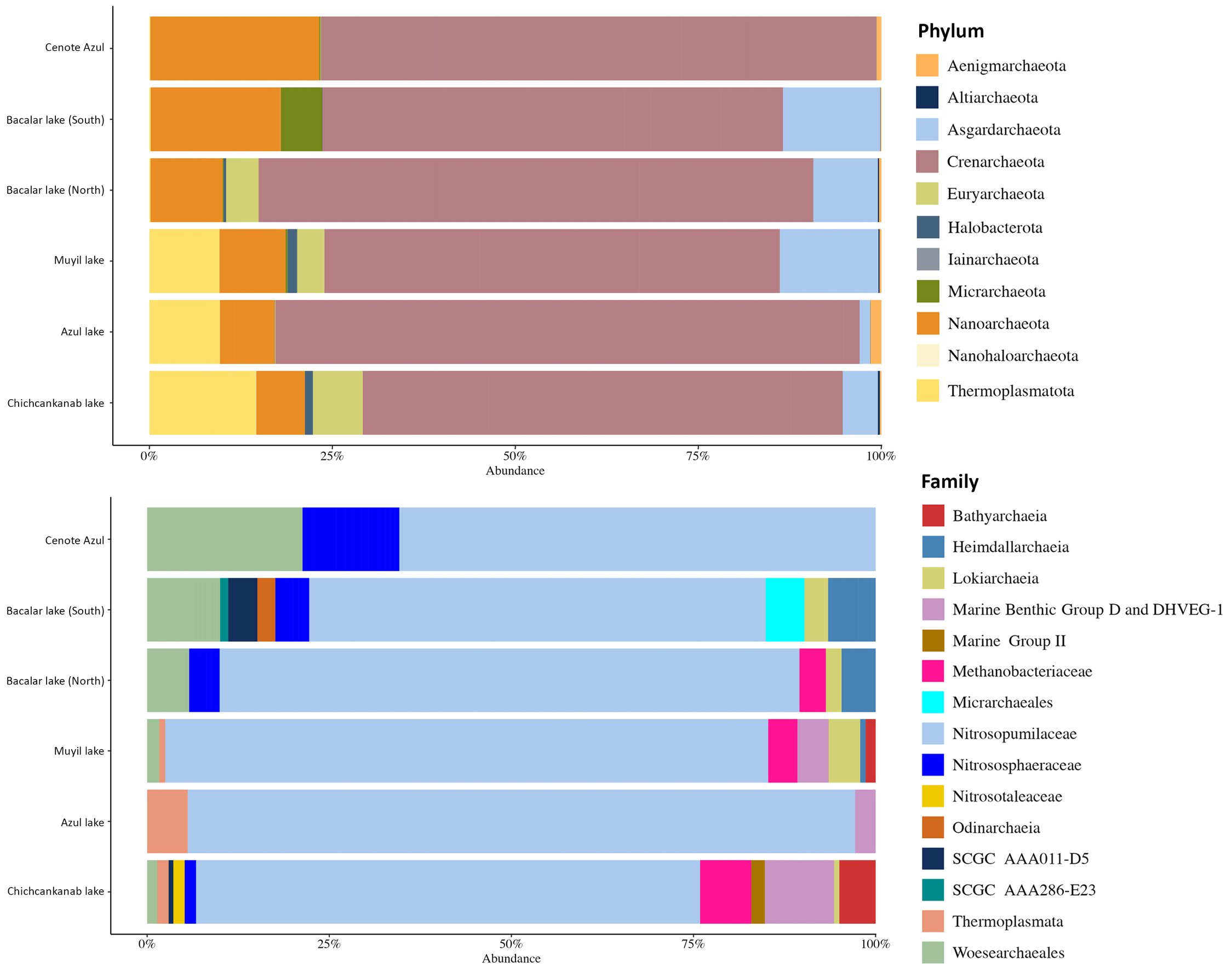

Supplement: S1 Fig — (TIF) [file pone.0322625.s001.tif]

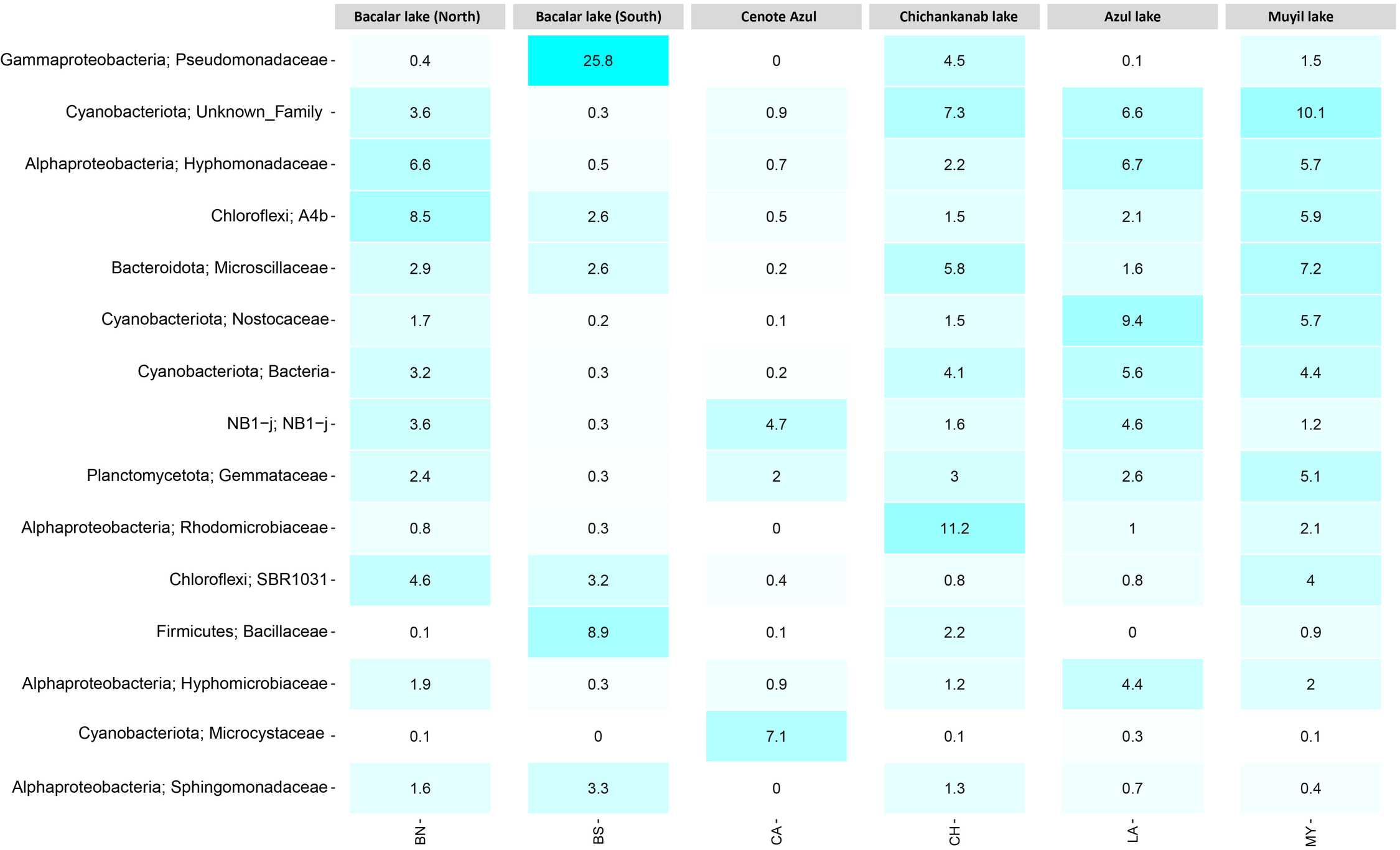

Supplement: S2 Fig — (TIF) [file pone.0322625.s002.tif]

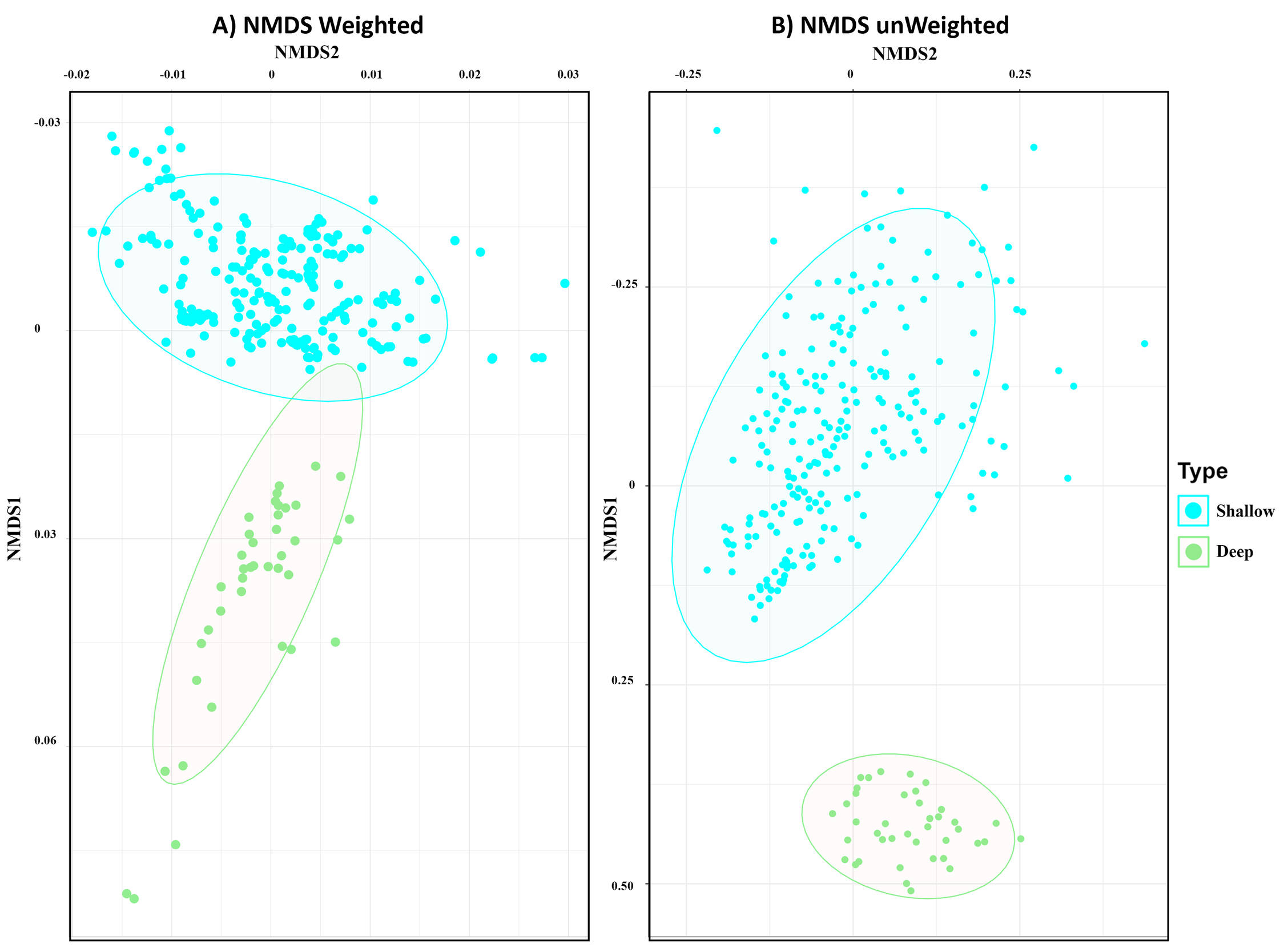

Supplement: S3 Fig — (TIF) [file pone.0322625.s003.tif]
