# Supplementary material for: Karst-environments of the southeastern Yucatan Peninsula: Hotspots for modern freshwater microbialites
Source: PLoS One. 2025 May 7;20(5):e0322625. doi: 10.1371/journal.pone.0322625 (PMC12057922; doi:10.1371/journal.pone.0322625)
Supplement: S1 Table — (DOCX) [file pone.0322625.s004.docx]

**S1 Table.** Location and characteristics of each study site.

| **Location** | **Latitude** | **Longitude** | **Geology**  **(Lithology, Ga, Pf)** | **LUC (1993-2018)** | **Neighboring localities**  **(potential LUC drivers)** |
| --- | --- | --- | --- | --- | --- |
| Chichancanab lake | 19.917372 | -88.77197 | Holocene sedimentary lacustrine lithology immersed in Eocene sedimentary Limestone-Marl. | Previously disturbed environments, however there is a high impact due to the expansion of the agricultural frontier (AF). | Dziuché |
| Azul lake | 19.884594 | -88.083401 | Pliocene sedimentary limestone-coquina. Nearby fault | High impact of AF expansion | Señor |
| Muyil lake | 20.062009 | -87.592336 | Holocene sedimentary lagoon | Less disturbed environment, increased mangrove coverage, moderate urbanization | Muyil |
| Bacalar lake North | 18.874423 | -88.237078 | Pliocene sedimentary limestone-coquina. Associated with geological fault | Growth of urban sprawl | Buenavista |
| Bacalar lake South | 18.570603 | -88.443977 | Holocene sedimentary alluvium. Associated with geological fault | High impact due to expansion of AF and urbanization in areas not immediately adjacent. | Xul Ha, Juan Sarabia, Ucum and others belonging to the local agricultural corridor Sergio Butrón Casas |
| Cenote Azul | 18.647555 | -88.41257 | Miocene sedimentary limestone. Associated with geological fault | Growth of urban sprawl and expansion of AF | Bacalar, Mennonite community of Salamanca |

**SOURCES**

Geological-Mining Chart 1:250,000 of the Mexican Geological Service (2015).

Land use and vegetation charts, series II (1993) and VII (2018) INEGI. Scale 1:250,000

Geomedian Landsat INEGI (https://www.inegi.org.mx/investigacion/geomediana/). 1993 to 2022

**Abbreviation**: Ga: Geological age; Pf: Presence faults; LUC: Land Use Change
