# Supplementary material for: Karst-environments of the southeastern Yucatan Peninsula: Hotspots for modern freshwater microbialites
Source: PLoS One. 2025 May 7;20(5):e0322625. doi: 10.1371/journal.pone.0322625 (PMC12057922; doi:10.1371/journal.pone.0322625)
Supplement: S2 Table — (DOCX) [file pone.0322625.s005.docx]

**S2 Table.** Alpha diversity indexes of microbial communities associated with microbialites in the lakes of Quintana Roo.

| **Location** | **Chao1** | **Shannon** | **InvSimpson** |
| --- | --- | --- | --- |
| Chichankanab lake | 346 | 4.8 | 35.13 |
| Azul lake | 1229 | 5.72 | 61.19 |
| Muyil lake | 924 | 5.49 | 63.79 |
| Bacalar lake North | 818 | 5.75 | 113.37 |
| Bacalar lake South | 277 | 3.37 | 9.65 |
| CenoteAzul | 1720 | 6.3 | 125.73 |
| **Kruskal–Wallis (*p-value*)** | <0.05 | <0.05 | <0.05 |
