# Supplementary material for: Karst-environments of the southeastern Yucatan Peninsula: Hotspots for modern freshwater microbialites
Source: PLoS One. 2025 May 7;20(5):e0322625. doi: 10.1371/journal.pone.0322625 (PMC12057922; doi:10.1371/journal.pone.0322625)
Supplement: S3 Table — (DOCX) [file pone.0322625.s006.docx]

**S3 Table.** Permutational multivariate analysis of variance (PERMANOVA) of microbial communities associated with microbialites (a); between shallow and deep sites (b).

|  | **Anosim** | | | **Adonis** | **betadisper** |
| --- | --- | --- | --- | --- | --- |
|  | | **R** | ***p-value*** | ***p-value*** | **R2** |
| 1. **All locations** | |  |  |  |  |
| weighted matrix | | 0.27 | 0.001 | 0.001 | 0.94 |
| unweighted matrix | | 0.22 | 0.001 | 0.001 | 0.91 |
|  | |  |  |  |  |
| b) shallow versus deep | |  |  |  |  |
| weighted matrix | | 0.30 | 0.001 | 0.001 | 0.77 |
| unweighted | | 0.089 | 0.001 | 0.001 | 0.74 |
| **matrix** | |  |  |  |  |
