# Supplementary material for: Karst-environments of the southeastern Yucatan Peninsula: Hotspots for modern freshwater microbialites
Source: PLoS One. 2025 May 7;20(5):e0322625. doi: 10.1371/journal.pone.0322625 (PMC12057922; doi:10.1371/journal.pone.0322625)
Supplement: S4 Table — (DOCX) [file pone.0322625.s007.docx]

**S4 Table.** LCBD *p-values* of the sites with microbialites in Quintana Roo.

| BDtotal | 0.63 |
| --- | --- |
| Sstotal | 3.19 |
| p.LCBD |  |
| Chichancanab lake | 1 |
| Azul lake | 1 |
| Muyil lake | 1 |
| Bacalar lake North | 1 |
| Bacalar lake South | 0.001 |
| CenoteAzul | 0.001 |
