# Supplementary material for: Karst-environments of the southeastern Yucatan Peninsula: Hotspots for modern freshwater microbialites
Source: PLoS One. 2025 May 7;20(5):e0322625. doi: 10.1371/journal.pone.0322625 (PMC12057922; doi:10.1371/journal.pone.0322625)
Supplement: S5 Table — (DOCX) [file pone.0322625.s008.docx]

**S5 Table.** Main species that contribute to the turnover of prokaryotic communities in microbialites of Quintana Roo.

| **Domain** | **Phylum** | **Class** | **Order** | **Family** | **Genus** | **Species** | **Contribution** |
| --- | --- | --- | --- | --- | --- | --- | --- |
| Bacteria | Pseudomonadota | Alphaproteobacteria | Rhizobiales | Rhodomicrobiaceae | Rhodomicrobium | uncultured | 0.029 |
| Bacteria | Cyanobacteriota | Cyanobacteriia | Cyanobacteriotales | Nostocaceae | RivulariaPCC-7116 | --- | 0.023 |
| Bacteria | Pseudomonadota | Gammaproteobacteria | Pseudomonadales | Pseudomonadaceae | Pseudomonas | --- | 0.023 |
| Bacteria | Pseudomonadota | Gammaproteobacteria | Pseudomonadales | Pseudomonadaceae | Pseudomonas | --- | 0.019 |
| Bacteria | Cyanobacteriota | Cyanobacteriia | Oxyphotobacteria | Unknown_Family | --- | --- | 0.015 |
| Bacteria | Bacteroidota | Bacteroidia | Cytophagales | Microscillaceae | --- | --- | 0.013 |
| Bacteria | Cyanobacteriota | Cyanobacteriia | Cyanobacteriotales | Microcystaceae | --- | --- | 0.013 |
| Bacteria | Pseudomonadota | Gammaproteobacteria | Aeromonadales | Aeromonadaceae | Aeromonas | uncultured | 0.010 |
| Bacteria | Cyanobacteriota | Cyanobacteriia | Cyanobacteriotales | Microcystaceae | --- | --- | 0.009 |
| Bacteria | Bacteroidota | Bacteroidia | Cytophagales | Microscillaceae | --- | --- | 0.008 |
| Bacteria | Cyanobacteriota | Cyanobacteriia | Oxyphotobacteria | Unknown_Family | --- | --- | 0.007 |
| Bacteria | Cyanobacteriota | Cyanobacteriia | Phormidesmiales | Phormidesmiaceae | PhormidiumMBIC10003 | --- | 0.007 |
| Bacteria | Pseudomonadota | Gammaproteobacteria | Xanthomonadales | Xanthomonadaceae | Stenotrophomonas | --- | 0.006 |
| Bacteria | Pseudomonadota | Gammaproteobacteria | Pseudomonadales | Moraxellaceae | Acinetobacter | --- | 0.005 |
| Archaea | Crenarchaeota | Nitrososphaeria | Nitrosopumilales | Nitrosopumilaceae | Nitrosopumilaceae | uncultured | 0.005 |
| Bacteria | Pseudomonadota | Alphaproteobacteria | Caulobacterales | Hyphomonadaceae | uncultured | uncultured | 0.005 |
| Bacteria | Methylomirabilota | Methylomirabilia | Methylomirabilales | Methylomirabilaceae | wb1-A12 | uncultured | 0.005 |
| Bacteria | Cyanobacteriota | Cyanobacteriia | Cyanobacteriotales | Nostocaceae | ScytonemaVB-61278 | --- | 0.005 |
| Bacteria | Pseudomonadota | Alphaproteobacteria | Rhizobiales | Hyphomicrobiaceae | Hyphomicrobium | --- | 0.005 |
| Bacteria | Cyanobacteriota | Cyanobacteriia | Oxyphotobacteria | Unknown_Family | uncultured | --- | 0.004 |
