# Supplementary material for: Karst-environments of the southeastern Yucatan Peninsula: Hotspots for modern freshwater microbialites
Source: PLoS One. 2025 May 7;20(5):e0322625. doi: 10.1371/journal.pone.0322625 (PMC12057922; doi:10.1371/journal.pone.0322625)
Supplement: S6 Table — (DOCX) [file pone.0322625.s009.docx]

**S6 Table.** Ion abundance in water column for each study site harboring microbialites in Quintana Roo.

| Location | Hydrogeochemistry |
| --- | --- |
| Chichancanab lake | Sulfate> calcium> chlorine> magnesium> sodium> bicarbonate |
| Azul lake | Chlorine> sulfate> bicarbonate> sodium> calcium> magnesium |
| Muyil lake | Chlorine> bicarbonate> sodium> calcium> sulfate> magnesium |
| Bacalar lake North | Sulfate> calcium> chlorine> bicarbonate> sodium> magnesium |
| Bacalar lake South | Sulfate> calcium> bicarbonate> magnesium> chlorine> sodium |
| CenoteAzul | Sulfate> calcium> bicarbonate> magnesium> chlorine> sodium |
